# Supplementary material for: Genome-Wide Identification of a Maize Chitinase Gene Family and the Induction of Its Expression by Fusarium verticillioides (Sacc.) Nirenberg (1976) Infection
Source: Genes (Basel). 2024 Aug 17;15(8):1087. doi: 10.3390/genes15081087 (PMC11353892; doi:10.3390/genes15081087)
Supplement: Supplementary file 1 [file genes-15-01087-s001.zip › Supplementary Figure S1.pdf]

In silico expression analysis of chitinase genes using RNA-seq data

In silico gene transcription analysis of the maize glycoside hydrolase family was performed using RNA-seq data in order to retrieve the expression data in response to abiotic stress. The fragment per kilobase of exon per million fragments mapped (FPKM) was obtained from the B73 V5 genome through a gene ID (Zm00001) search using qTeller in the MaizeGDB database [36]. Previous reports were used to study gene expression for abiotic stress using seedling [37] and leaf [38] samples. The extracted data were then log2 transformed and used to generate heatmaps via the TBtools package [39].

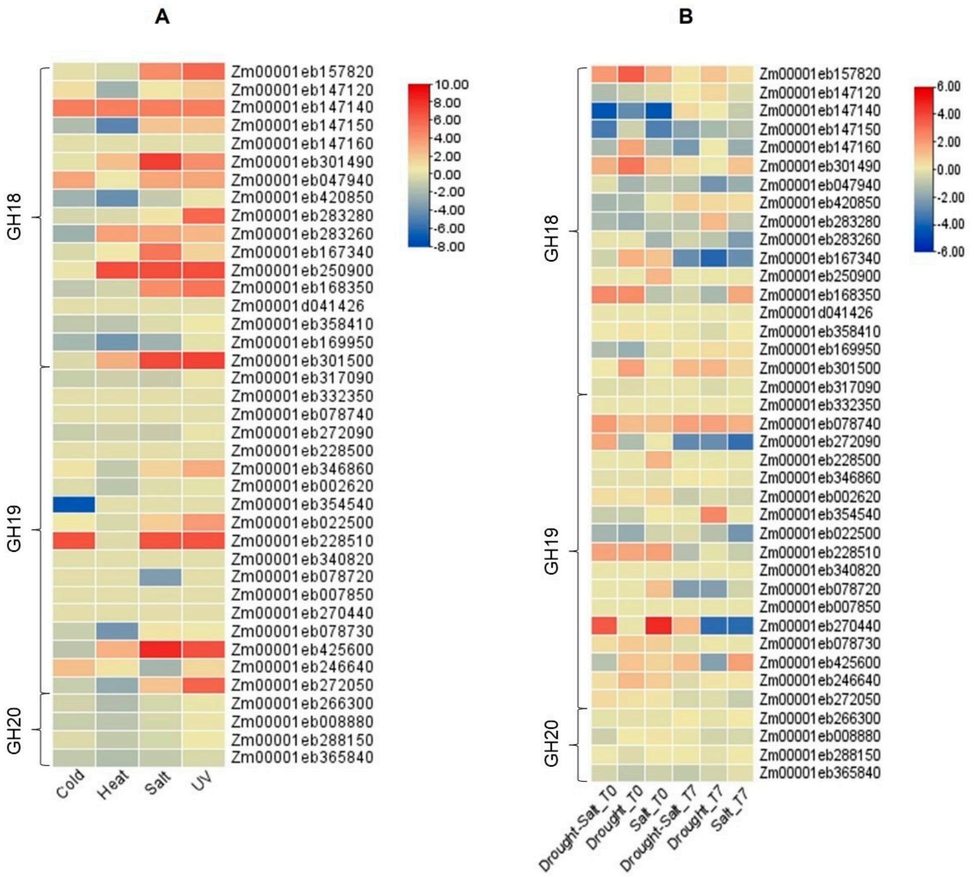

Supplementary Figure S1. Heat map of chitinase mRNA expression in *Zea mays*. (a) Maize chitinase gene expression in seedling samples; (b) maize chitinase gene expression in leaf samples. The color range indicates a gene expression gradient from repressed (blue) to induced (red) expression relative to control untreated plants.
